# Association of Aortic Cross-Clamping Time with Systemic Immune Inflammation and Systemic Inflammatory Response Indexes in Isolated Coronary Bypass Surgery

**DOI:** 10.21470/1678-9741-2024-0266

**Published:** 2025-09-11

**Authors:** Duygu Durmaz, Sedat Gündöner, Hayrettin Tekümit

**Affiliations:** 1 Department of Cardiothoracic and Vascular Surgery, Bandirma Onyedi Eylül University, Balıkesir, Turkiye

**Keywords:** Coronary Artery Bypass, Constriction, Neutrophils, Inflammation, Biomarkers, Lymphocytes, Systemic Inflammatory Response Syndrome.

## Abstract

**Introduction:**

Prolonged aortic cross-clamping may intensify systemic inflammation after cardiac surgery. This study aimed to evaluate the effect of cross-clamp duration on systemic inflammatory response index (SIRI) and systemic immune inflammation index (SIII) in isolated coronary artery bypass grafting (CABG).

**Method:**

This retrospective study included 155 patients who underwent first-time isolated CABG between January 2021 and June 2024. Patients were divided into two groups based on median cross-clamping time: Group I (≤ 64 minutes, n = 83) and Group II (> 64 minutes, n = 72). Demographic, hematologic, and biochemical data were collected. SIII was calculated as platelet × neutrophil/lymphocyte; SIRI as neutrophil × monocyte/lymphocyte.

**Results:**

The mean aortic cross-clamping time of Group I was 53 minutes (interquartile range 44 - 60 minutes) and of Group II it was 78 minutes (interquartile range 71 - 87 minutes) (P < 0.001). An increase in systemic immune inflammation index and systemic inflammatory response index values was observed in both groups at the 24^th^ postoperative hour. Postoperative systemic immune inflammation index and systemic inflammatory response index levels were significantly higher in Group II (P < 0.05). There was a weak but significant positive correlation between aortic cross-clamping time and postoperative systemic inflammation response index (r = 0.220; P = 0.006).

**Conclusion:**

Prolonged aortic cross-clamping time is associated with an increased postoperative inflammatory response. These indices may serve as biomarkers for evaluating systemic inflammation following coronary artery bypass grafting.

## INTRODUCTION

**Table:** 

Abbreviations, Acronyms & Symbols				
ACC	= Aortic cross-clamping		EuroSCORE	= European System for Cardiac Operative Risk Evaluation
BMI	= Body mass index		Hb	= Hemoglobin
BSA	= Body surface area		SD	= Standard deviation
CABG	= Coronary artery bypass grafting		SE	= Standard error
CI	= Confidence interval		SIII	= Systemic immune inflammation index
CPB	= Cardiopulmonary bypass		SIRI	= Systemic inflammatory response index
CRP	= C-reactive protein		SIRS	= Systemic inflammatory response syndrome
EF	= Ejection fraction			

During cardiopulmonary bypass (CPB), patients receive continuous blood flow through a mechanical pump, while aortic cross-clamping (ACC) is applied to ensure a motionless and bloodless surgical field^[1,2]^. However, CPB and ACC may trigger systemic inflammatory response syndrome (SIRS), characterized by the release of various pro-inflammatory mediators, which can adversely affect postoperative outcomes^[3]^.

SIRS may be significantly increased following coronary artery bypass grafting (CABG) due to the duration of CPB and ACC. Previous studies have demonstrated that prolonged CPB and ACC times are predictors of increased mortality and morbidity^[4,5]^. However, there is no consensus regarding a safe duration limit for these procedures, and different studies have reported varying results.

In recent years, systemic inflammatory response index (SIRI) and systemic immune inflammation index (SIII) have been introduced as novel inflammatory biomarkers^[6,7]^. These indices incorporate key inflammatory components, including neutrophils, monocytes, lymphocytes, and platelets, and have been suggested as potential prognostic markers in various cardiovascular diseases^[8,9]^. While these indices have been studied in different clinical conditions, limited data exists regarding their relationship with ACC time during CABG.

This study aimed to evaluate the relationship between ACC time and inflammatory markers (SIRI and SIII) in isolated CABG. Understanding this association may help identify patients at higher risk of excessive postoperative inflammation, potentially guiding early intervention strategies to optimize postoperative care.

## METHODS

### Study Design and Patients

The population of this retrospective study consisted of 155 patients undergoing first-time isolated CABG at a single center between January 2021 and June 2024. Patients over 18 years of age who underwent elective isolated on-pump CABG were included. Reoperations, patients with additional surgical procedures, patients with known cerebrovascular disease, bleeding pathology, and patients with missing medical data were excluded. The study population was divided into two groups according to the median ACC duration (64.0 minutes) of the total study population: Group I (n = 83) with ACC duration of ≤ 64 minutes and Group II (n = 72) with ACC duration of ≥ 64 minutes.

**Fig. 1:**
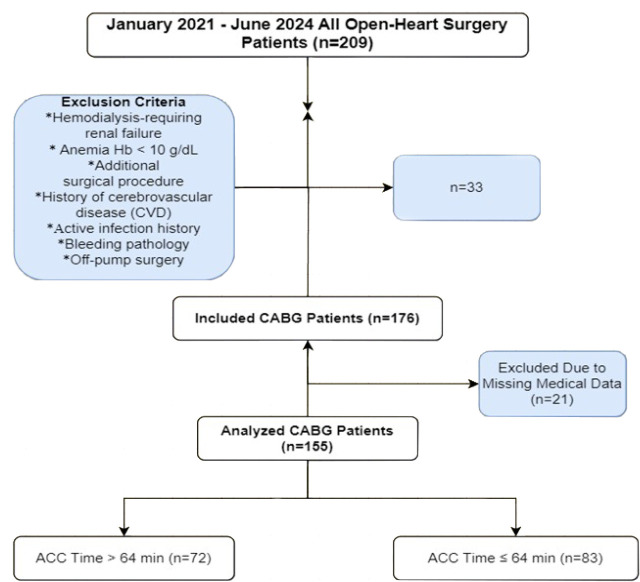
Flowchart of the study. ACC=aortic cross-clamping; CABG=coronary artery bypass grafting; Hb=hemoglobin.

All procedures performed in studies involving human participants were in accordance with the ethical standards of the institutional research committee and the 1964 Declaration of Helsinki and ethical standards. The study was approved by the Bandirma Onyedi Eylül University Non-Interventional Research Ethics Committee with the ethics committee decision dated 16.01.2024 and numbered 2024-1. The flow diagram of the study is shown in Figure 1.

### Data Collection and Calculation of Inflammatory Indexes

Demographic data, preoperative and postoperative hematologic and biochemical analyses, and clinical data including postoperative processes were obtained by examining the computerized patient record system. SIRI and SIII related with systemic inflammation were calculated as follows^[10]^: SIII = peripheral platelet count × neutrophil count/lymphocyte count; and SIRI = neutrophil count × monocyte count/lymphocyte count.

### Operative Technique

All patients underwent standard median sternotomy under general anesthesia. After appropriate anticoagulation with 400 IU/kg heparin, CPB was initiated by cannulation of the ascending aorta and right atrial cannulation. All patients were monitored with moderate hypothermia (28 - 32 °C), and myocardial protection was achieved with antegrade-retrograde combined blood cardioplegia. Mean arterial pressure was maintained between 60 and 80 mmHg during CPB. After the procedure was completed, CPB was terminated as soon as the body temperature of the patients was raised to 37 °C. Decannulation was performed after neutralization with protamine.

### Statistical Analysis

Statistical evaluation was performed with IBM SPSS Statistics for Windows, version 25.0 (IBM Corp., Armonk, NY., USA) package program. While evaluating the study data, quantitative variables were represented by mean, standard deviation, median, and minimum and maximum values, and qualitative variables were represented by descriptive statistical methods such as frequency and percentage. Shapiro-Wilks test and Box Plot graphs were used to evaluate the conformity of the data to normal distribution. Student's *t*-test was used for quantitative two-group evaluations showing normal distribution, and Mann-Whitney U test was used for those not showing normal distribution. Between two follow-ups, paired samples test was used for those with normal distribution, and Wilcoxon test was used for those without normal distribution. Chi-square test and Fisher’s exact test were used to compare qualitative data. Pearson correlation analysis was used to evaluate the relationships between variables. The results were evaluated at 95% confidence interval, and significance was evaluated at *P* < 0.05 level.

## RESULTS

A total of 155 patients who underwent isolated CABG were included in the study; 63.9% of patients in Group I and 75% of patients in Group II were male. There were no statistically significant differences in age, body mass index, body surface area, ejection fraction, and European System for Cardiac Operative Risk Evaluation II values. However, there was a significant difference in the duration of CPB and ACC and the number of CABGs between the groups (Table 1).

**Table 1:** Demographic and operative data of the patients (n = 155).

Variables	Group 1 (n = 83)	Group 2 (n = 72)	*P*-value
Sex (male)	53 (63.9%)	54 (75%)	1.00^a^
Age	64 (59 - 69)	67 (62 - 73)	0.053^b^
BMI	28.7 (26.4 - 29.7)	28.7 (25.8 - 30.4)	0.827^b^
BSA (m^2^)	1.87 (1.76 - 1.98)	1.91 (1.81 - 2.04)	0.033^b^
EF (%)	50 (45 - 50)	50 (45 - 50)	0.109^b^
EuroSCORE II	6.2 (5.28 - 8.30)	6.9 (5.8 - 9)	0.109^b^
CPB (min.)	102 (93 - 118)	129.5 (118.5 - 143.7)	< 0.001^b^*^^
ACC (min.)	53 (44 - 60)	78 (71 - 87)	< 0.001^b^*^^
CABG (count)	3 (2 - 3)	3 (3 - 4)	< 0.001^b^*^^

a Fisher's exact or χ^^2^^ tests;

b Mann-Whitney U test;

* *P* < 0.001; Data expressed as median (25^th^ to 75^th^ percentiles)

ACC=aortic cross-clamping; BMI=body mass index; BSA=body surface area; CABG=coronary artery bypass grafting; CPB=cardiopulmonary bypass; EF=ejection fraction; EuroSCORE=European System for Cardiac Operative Risk Evaluation

**Fig. 2:**
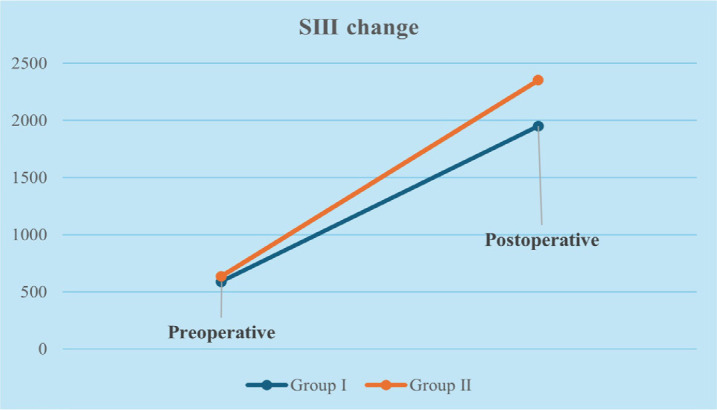
Systemic immune inflammation index (SIII) change according to groups.

**Fig. 3:**
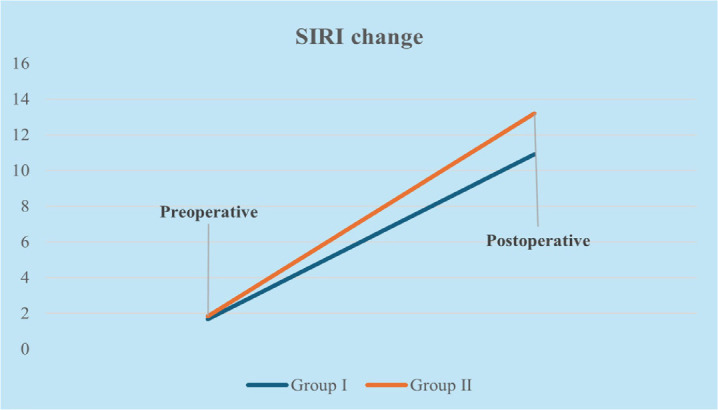
Systemic inflammatory response index (SIRI) change according to groups.

The changes in inflammatory indices and biochemical markers within and between groups at preoperative and postoperative 24-hour periods are shown in Table 2. In both groups, a significant increase was observed in postoperative SIII, SIRI, C-reactive protein (CRP), and a significant decrease in albumin levels compared to preoperative values (*P* < 0.005). When comparing the groups, preoperative levels of CRP, SIII, SIRI, and albumin were similar, whereas postoperative SIII and SIRI values were significantly higher in Group II (*P* < 0.005) (Table 2). The graphical changes of SIII and SIRI parameters of Group I and Group II are shown in Figures 2 and 3.

**Table 2:** Preoperative and postoperative parameters (n = 155).

		Groups		*P*-value
		Group 1 (n = 83)	Group 2 (n = 72)	
SIII				
Preoperative	Median (25 - 75 per.)	588.1 (420 - 827.2)	634 (441.5 - 828.3)	0.672^a^
Postoperative	Median (25 - 75 per.)	1.951 (1.366 - 2.776)	2.351 (1.688 - 3.301)	0.042^a^
	*P*-value^b^	< 0.001^*^	< 0.001^*^	
SIRI				
Preoperative	Median (25 - 75 per.)	1.66 (1.17 - 2.7)	1.84 (1.35 - 2.31)	0.845^a^
Postoperative	Median (25 - 75 per.)	10.9 (8.5 - 14.6)	13.2 (9.6 - 21.5)	0.011^a^
	*P*-value^b^	< 0.001^*^	< 0.001^*^	
Albumin				
Preoperative	Median (25 - 75 per.)	4 (3.7 - 4.2)	4 (3.8 - 4.3)	0.363^a^
Postoperative	Median (25 - 75 per.)	3.4 (3.2 - 3.7)	3.4 (3.2 - 3.5)	0.193^a^
	*P*-value^b^	< 0.001^*^	< 0.001^*^	
CRP				
Preoperative	Median (25 - 75 per.)	1.5 (0.6 - 2.4)	1.1 (0.4 - 2)	0.064^a^
Postoperative	Mean (SD)	8.9 ± 2.8	8.5 ± 3.2	0.547^c^
	*P*-value^b^	< 0.001^*^	< 0.001^*^	

a Mann-Whitney U test;

b Wilcoxon rank test;

c Student’s *t*-test;

* *P* < 0.001; Data expressed as median (25^th^ to 75^th^ percentiles)

CRP=C-reactive protein; SD=standard deviation; SIII=systemic immune inflammation index; SIRI=systemic inflammatory response index

When the correlation of SIRI and SIII values with ACC duration, postoperative albumin, and CRP values was examined, a weak but statistically significant positive correlation was observed between the duration of ACC and postoperative SIRI (r = 0.220; *P* = 0.006) and postoperative SIII (r = 0.168; *P* = 0.037). In addition, a positive, weak but significant correlation was found between postoperative albumin and postoperative SIII (Table 3). No statistically significant correlation was found for other postoperative variables.

**Table 3:** Correlation of variables (n = 155).

Variables		Postoperative SIII	Postoperative SIRI	Postoperative albumin	Postoperative CRP
ACC duration	r	0.168	0.220	-0.046	-0.034
	*P*-value	0.037^*^	0.006^*^	0.567	0.670
Postoperative albumin	r	0.182	0.164	-	-0.058
	*P*-value	0.023^*^	0.042	-	0.477
Postoperative CRP	r	0.095	0.068	-0.058	-
	*P*-value	0.240	0.404	0.477	-

* Spearman correlation

ACC=aortic cross-clamping; CRP=C-reactive protein; SIII=systemic immune inflammation index; SIRI=systemic inflammatory response index

The prediction of postoperative SIRI index by ACC duration was analyzed with a simple linear regression model. The model created as a result of the analysis was statistically significant (F = 7.455; *P* = 0.007). The ACC duration of the patients explained 4.6% of the variance in postoperative SIRI (R^2^ = 0.046). Each one-minute increase in ACC time was associated with a 0.098-unit increase in postoperative SIRI levels (Table 4).

**Table 4:** Effect of ACC on postoperative SIRI index.

Independent variables	Unstandardized coefficients		Standardized coefficients	t	*P*-value	95% CI
	B	SE	β			
(Constant)	8.052	2.421		3.326	0.001	3.269 - 12.835
ACC	0.098	0.036	0.216	2.730	0.007	0.027 - 0.169

Dependent variable: postoperative SIRI index; β: standardised regression coefficient; Durbin-Watson = 1.701; F = 7.455; *P* = 0.007; R2 = 0.046

ACC=aortic cross-clamping; CI=confidence interval; SE=standard error; SIRI=systemic inflammatory response index

## DISCUSSION

To our knowledge, this is the first study investigating the relationship between ACC time and inflammatory indices in patients undergoing isolated on-pump CABG. Previous studies have reported that prolonged ACC time is associated with increased complications and mortality risk in cardiac surgery^[11-13]^. However, there is limited data on how systemic inflammatory response indices, such as SIII and SIRI, change in relation to ACC duration.

The SIII index, incorporating neutrophils, lymphocytes, and platelets, has been identified as a prognostic marker in various cardiovascular conditions, including coronary revascularization^[14]^. It has also been linked to inflammatory processes in conditions such as acute appendicitis and malignancies^[15-17]^. Similarly, SIRI, which includes neutrophils, monocytes, and lymphocytes, is an emerging inflammatory index associated with systemic immune response and disease severity in different clinical settings^[7,18]^.

CPB is widely used to facilitate cardiac surgery but has been associated with SIRS due to blood contact with artificial surfaces, activation of coagulation and complement systems, endothelial dysfunction, and platelet activation^[19-21]^. Prolonged CPB and ACC further amplify these inflammatory responses and are linked to worse postoperative outcomes, including increased morbidity and decreased early survival^[22-24]^.

In our study, patients were categorized based on the median ACC time (64 minutes). While baseline demographic characteristics and preoperative inflammatory indices were similar between groups, postoperative SIII and SIRI values were significantly higher in the group with prolonged ACC. This suggests that extended ischemic time. contributes to an increased inflammatory response. These findings align with previous studies showing that prolonged CPB is associated with increased neutrophil activation and systemic inflammation^[25]^.

CRP, a widely used inflammatory biomarker, has been shown to increase significantly after cardiac surgery involving CPB^[26]^. In our study, while postoperative CRP levels were significantly elevated compared to preoperative values in both groups, there was no significant difference between the groups. Similarly, serum albumin levels, which decrease due to hemodilution and inflammation, showed a significant decline postoperatively but remained comparable between groups^[27,28]^.

The main contributors to inflammation during CABG include surgical stress, aortic manipulation, ACC, pericardial aspiration, and internal mammary artery harvesting. Our findings indicate that SIRI and SIII indices correlate positively with prolonged ACC, with SIRI showing a stronger association. These indices may serve as useful biomarkers for assessing postoperative inflammatory responses, complementing traditional markers such as CRP and white blood cell count.

Clinically, monitoring these indices may help identify patients at higher risk of excessive inflammatory responses following CABG. Early recognition of patients with elevated SIRI and SIII levels could facilitate targeted anti-inflammatory strategies, such as optimized CPB management, modified myocardial protection strategies, or pharmacological interventions to mitigate inflammatory responses.

Our study demonstrates that prolonged ACC time is associated with an increased postoperative inflammatory response. SIRI and SIII indices may serve as additional biomarkers for assessing inflammation in patients undergoing isolated CABG, potentially aiding in risk stratification and personalized postoperative management.

### Limitations

The limitations of this study include its retrospective design, the relatively small sample size, and the fact that it was conducted at a single center, which may limit the generalizability of the findings.

## CONCLUSİON

Our study is the first to evaluate the relationship between inflammatory indices and duration of surgery in patients undergoing isolated CABG surgery. The significant increase in SIRI and SIII levels with longer ACC times emphasizes that these indices are potential markers of inflammatory response in cardiac surgery. Despite some limitations, our results suggest that monitoring these indices may improve postoperative management and outcomes in CABG patients. We recommend further research with larger, randomised cohorts to confirm these findings.
